# Discovery of Potential Noncovalent Inhibitors of Dehydroquinate Dehydratase from Methicillin-Resistant *Staphylococcus aureus* through Computational-Driven Drug Design

**DOI:** 10.3390/ph16081148

**Published:** 2023-08-12

**Authors:** César Millán-Pacheco, Lluvia Rios-Soto, Noé Corral-Rodríguez, Erick Sierra-Campos, Mónica Valdez-Solana, Alfredo Téllez-Valencia, Claudia Avitia-Domínguez

**Affiliations:** 1 Facultad de Farmacia, Universidad Autónoma del Estado de Morelos, Cuernavaca, Morelos 62209, Mexico; cmp@uaem.mx; 2 Facultad de Medicina y Nutrición, Universidad Juárez del Estado de Durango, Av. Universidad y Fanny Anitua S/N, Durango 34000, Mexico; lluviarios.soto@gmail.com (L.R.-S.); noe.corral.96@outlook.com (N.C.-R.); 3 Facultad de Ciencias Químicas, Universidad Juárez del Estado de Durango Campus Gómez Palacio, Avenida Artículo 123 S/N, Fracc. Filadelfia, Gómez Palacio 35010, Mexico; ericksier@gmail.com (E.S.-C.); valdezandyval@gmail.com (M.V.-S.)

**Keywords:** MRSA, shikimate pathway, dehydroquinate dehydratase, virtual screening, molecular dynamics, computer-aided drug design

## Abstract

Bacteria resistance to antibiotics is a concerning global health problem; in this context, methicillin-resistant *Staphylococcus aureus* (MRSA) is considered as a high priority by the World Health Organization. Furthermore, patients with a positive result for COVID-19 received early antibiotic treatment, a fact that potentially encourages the increase in antibiotic resistance. Therefore, there is an urgency to develop new drugs with molecular mechanisms different from those of the actual treatments. In this context, enzymes from the shikimate pathway, a route absent in humans, such as dehydroquinate dehydratase (DHQD), are considered good targets. In this work, a computer-aided drug design strategy, which involved exhaustive virtual screening and molecular dynamics simulations with MM-PBSA analysis, as well as an in silico ADMETox characterization, was performed to find potential noncovalent inhibitors of DHQD from MRSA (SaDHQD). After filtering the 997 million compounds from the ZINC database, 6700 compounds were submitted to an exhaustive virtual screening protocol. From these data, four molecules were selected and characterized (**ZINC000005753647** (**1**), **ZINC000001720488** (**2**), **ZINC000082049768** (**3**), and **ZINC000644149506** (**4**)). The results indicate that the four potential inhibitors interacted with residues important for substrate binding and catalysis, with an estimated binding free energy like that of the enzyme’s substrate. Their ADMETox-predicted properties suggest that all of them support the structural characteristics to be considered good candidates. Therefore, the four compounds reported here are excellent option to be considered for future in vitro studies to design new SaDHQD noncovalent inhibitors and contribute to the search for new drugs against MRSA.

## 1. Introduction

Bacterial resistance to antibiotics is a concerning global health problem [1] that is constantly evolving, where the emergence of antibiotic resistance is an outcome of a repertoire of factors in various environmental and clinical settings that have important repercussions on the health of the population [2,3,4]. Furthermore, increased exposure to healthcare and invasive procedures implies expanded antibiotic use, which further increases the risk for resistant pathogens to emerge [5]. Moreover, another situation is currently taking place; reports are emerging that pose a concerning situation; the intensity with which the COVID-19 pandemic is affecting healthcare and community environments is threatening [6,7,8]. High volumes of patients are becoming infected over short periods of time, resulting in a spike in antimicrobial consumption. According to a review of the data on COVID-19 cases, approximately 70% of patients with a positive result of COVID-19 received early antibiotic treatment when only approximately 8% needed it [9,10], a fact that could potentially encourage the increase of antibiotic resistance.

Even though effective antibiotics are a cornerstone of modern medicine, few bacteria can be as threatening as *Staphylococcus aureus*, a pathogen considered a high priority by the World Health Organization [11,12] and implicated in skin and soft tissue infections. One of the principal challenges is the methicillin-resistant *S. aureus* (MRSA) strains, because this pathogen is resistant to most of the actual treatments, which in turn results in high rates of mortality [13]. Therefore, new treatments are required to be able to manage this increasing threat. Under this light, a fundamental metabolic pathway for bacteria’s survival is the Shikimate Pathway (SK), a biosynthetic route that links carbohydrate metabolism through glycolysis and the pentose phosphate pathway, which has been considered an excellent target for antibacterial drug design, furthermore, this pathway is absent in humans [14]. This pathway consists of seven enzymatic steps that conclude with the formation of chorismate, a precursor of aromatic compounds, folates, and ubiquinone, as the final product [15,16].

One of these enzymes is 3-dehydroquinate dehydratase, which exists in two isoforms denoted as Type I and Type II. Structurally, Type I enzymes are homodimers, whilst Type II are dodecamers [17,18]. Type I 3-dehydroquinate dehydratase (DHQD) is encoded by the gene aroD and is found in fungi, plants, and bacteria, including the pathogenic *Salmonella* (*typhi* and *enterica*), *Escherichia coli*, *Clostridium difficile*, and *Staphylococcus aureus* [19,20,21]. DHQD catalyzes the reversible conversion of 3-dehydroquinate to 3-dehydroshikimate by a syn-elimination of water, another difference with Type II, which performs it through an Anti-elimination of water [22,23].

In addition to the biosynthetic role of DHQD, deletions in the aroD gene in *S. typhi*, *S. flexneri*, and *S. auerus* have suggested that this enzyme may act as a virulence factor [24]. In the same context, in *S. aureus*, a Small Colony Variant (SCV) phenotype was isolated. This strain supports an Ochre mutation in the aroD gene, being auxotrophic for all aromatic amino acids and less virulent than the wild type [25]. Furthermore, aroD gene mutants have been proposed as vaccines for *Francisella tularensis*, the causative agent of Tularemia [26]. Therefore, this enzyme has been considered a promising target not only for antibiotic drug design but also for the design of new antivirulence drugs [24]. 

In this work, a computer-aided drug design strategy, including exhaustive virtual screening, molecular dynamics, MM/PBSA analysis, and in silico ADMETox predictions, was followed to find potential noncovalent inhibitors of DHQD from methicillin-resistant *Staphylococcus aureus* (SaDHQD). The molecules reported here potentially bind to the active site of the enzyme and support the structural characteristics to be considered promising drug candidates to fight against antibacterial resistance.

## 2. Results and Discussion

### 2.1. Database Filtering and Virtual Screening

**Figure 1:**
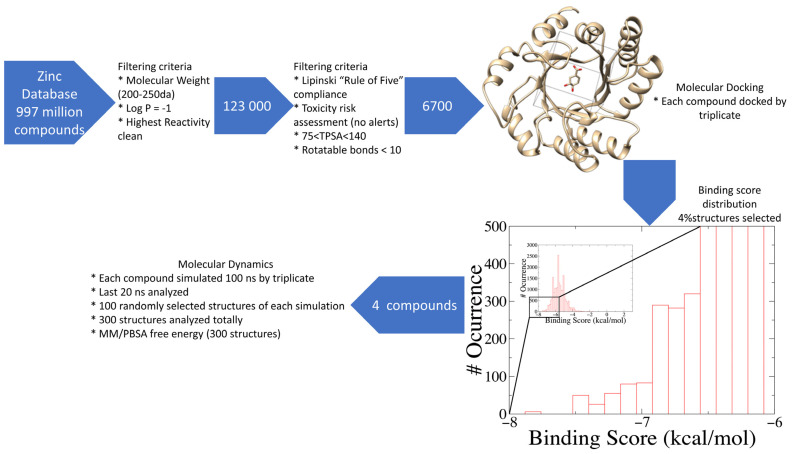
Workflow to obtain potential SaDHQD noncovalent inhibitors.

Nowadays, only a few covalent (irreversible) inhibitors of SaDHQD have been reported [27]. Noncovalent (reversible) inhibitors of DHQD have been reported only from other bacteria such as *C. difficile* and *E. faecalis* [28,29]. With the aim of obtaining the first potential noncovalent inhibitors from SaDHQD, a computer-aided drug design protocol was implemented (Figure 1). By the time the ZINC database was consulted, 997 million compounds were included in the TRANCHES 3D section. The first filtering criteria was to select structures with physicochemical characteristics similar to those of the substrate of the enzyme (3-dehydroquinate, MW = 198 Da and Log *p* = −1.7), therefore, compounds with a MW in the range of 200–250 Da, and a Log *p* value of −1 were included. Additionally, the highest reactivity clean criteria (without reactive groups) were applied, keeping only 123,000 molecules. Thereafter, Lipinski’s rule of five compliance, toxicity risk assessment, Topological Surface Area (TPSA), and rotatable bond filters were applied, leaving a final set of 6700 compounds. Nowadays, a lot of works using different virtual screening protocols to filter large chemical libraries have been published, but the challenge is always to select the best candidates for further experimental studies [30]. In this context, approximations such as consensus docking or rescoring functions have been used [31]. Here, with the aim to select molecules that potentially recognize the catalytic site of SaDHQD and to ensure that each result was consistent, three independent virtual screening assays over the same database (6700 compounds) were conducted using Autodock Vina (Table S1). It is important to mention that stereoisomers from compounds with chiral centers in their structure were included. The goal of this was to use molecular docking to find the compounds with the best binding pose according to their docking score for further computational characterization, not to perform binding free energy determinations [32]. The data show that four compounds met the requirement: **ZINC000005753647** (**1**), **ZINC000001720488** (**2**), **ZINC000082049768** (**3**), and **ZINC000644149506** (**4**) (Table 1). 

### 2.2. Molecular Dynamics Simulations

**Figure 2:**
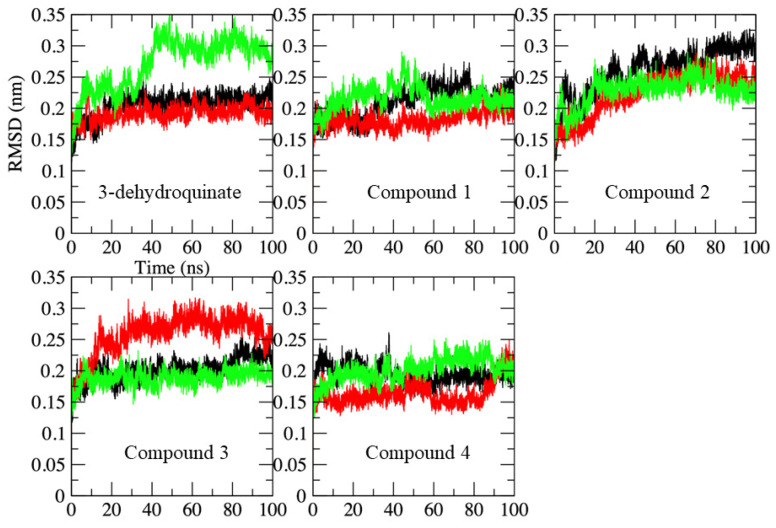
Alpha carbon-RMSD along the 100 ns of simulated time in each complex. Replicates are shown in different colors (black, replicate 1; red, replicate 2; and green, replicate 3). Axis labels are shown only on the first graph to avoid confusion.

At this moment, we have a set of possible compounds that might bind to SaDHQD. However, those ligands were selected using molecular docking that does not include sidechain and/or protein conformational changes that may influence the ligand’s binding to the enzyme [33]. To gain more information about the enzyme-ligand interaction, three independent 100 ns molecular dynamics simulations were conducted on each system, including the crystallographic ligand (3-dehydroquinate) (Figure 2). With the aim of performing the analysis on the period when the simulations were stable over time, the last 20 ns were used for the analysis of each trajectory. Results show that the α-carbon Root Mean Square Deviation (RMSD) for most of the systems simulated was stable along the last 20 ns, but there were a couple of systems where RMSD fluctuations indicate that they may not be stable or higher than the other replicas. However, fluctuations observed for those systems were lower than 3.5 Å when compared to all systems; in fact, all systems had a RMSD variation against the initial of no more than 3.5 Å (see, for example, the green line on the 3-dehydroquinate complex and compare it with the red line from compound 4 on Figure 2); therefore, those trajectories were included and analyzed. 

**Figure 3:**
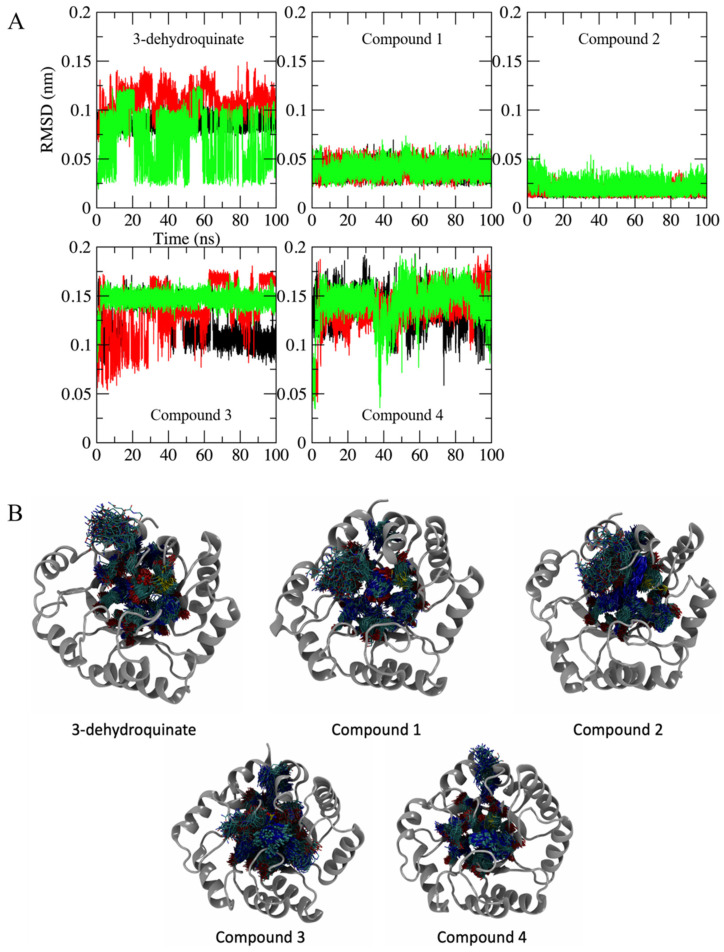
Ligand-RMSD. (**A**) RMSD along the 100 ns of simulated time in each complex. Replicates and axes labels are shown as described in Figure 2. (**B**) Ligand movement (brighter atoms at the center of each image) on the binding site (darker atoms on each image) in SaDHQD during the last 20 ns. The image also shows the movement of the lateral chains from the amino acids in the binding site.

The same situation was observed when the position of the ligands along the simulations was analyzed. In this case, 3-dehydroquinate shows the highest variations, followed by compounds **3** and **4**. However, the variations were so small that it can be said that all the ligands kept their binding sites. In fact, when ligand movement is observed in the binding site, complexes corresponding to substrate and compounds **3** and **4** show more variations (Figure 3). 

**Figure 4:**
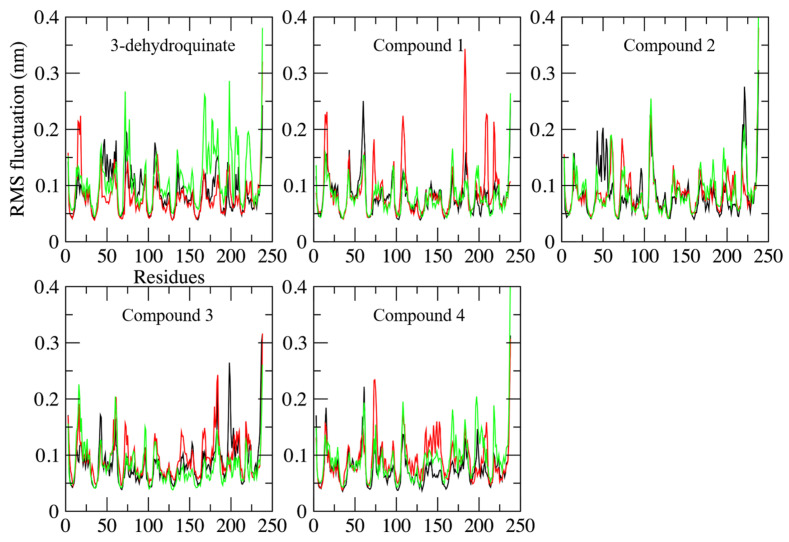
Root mean square fluctuation along the 100 ns of simulated time in each complex. Replicates and axes labels are shown as described in Figure 2.

On the other hand, no important changes were observed in Root Mean Square Fluctuations (RMSF) plots, which are used to explore the flexibility of each residue during simulations [34]. Therefore, the binding of the potential inhibitors did not provoke substantial alterations in the lateral chains of amino acids (Figure 4). 

**Figure 5:**
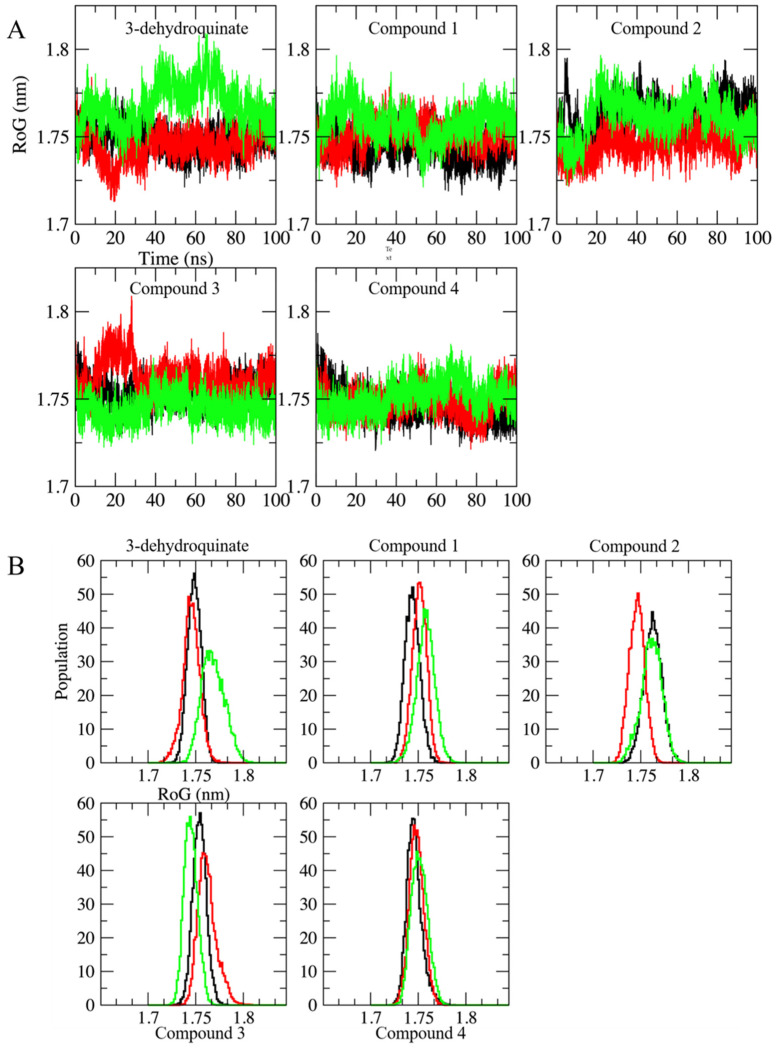
Radius of gyration along the 100 ns of simulated time in each complex. Replicates and axes labels are shown as described in Figure 2. (**A**) RoG of the systems herein simulated along 100 ns. (**B**) Normalized distribution of the RoG for all the systems.

This is in consonance with data obtained from Radius of Gyration (RoG) plots. RoG is a quantity that can be related to the natural protein “breathing”. A high value of RoG might be an indication of a possible denaturation of the protein; the opposite might be related to a compaction of the protein. Initial RoG was 1.72 ± 0.003 nm. RoG values over time show fluctuations with increments above 1.8 nm. These fluctuations might indicate that the protein had an opening that can be related to the natural movement of the amino or carboxyl terminal. However, RoG distributions show that (even in the worst case: 3-dehydroquinate simulation in green), these values are located around 1.76 and 1.77 nm. These values show an increment of 0.4 Å when compared to the initial value. Therefore, no effect was observed in the compactness of the protein structure, i.e., the binding of the compounds did not generate a crucial conformational change (Figure 5).

**Figure 6:**
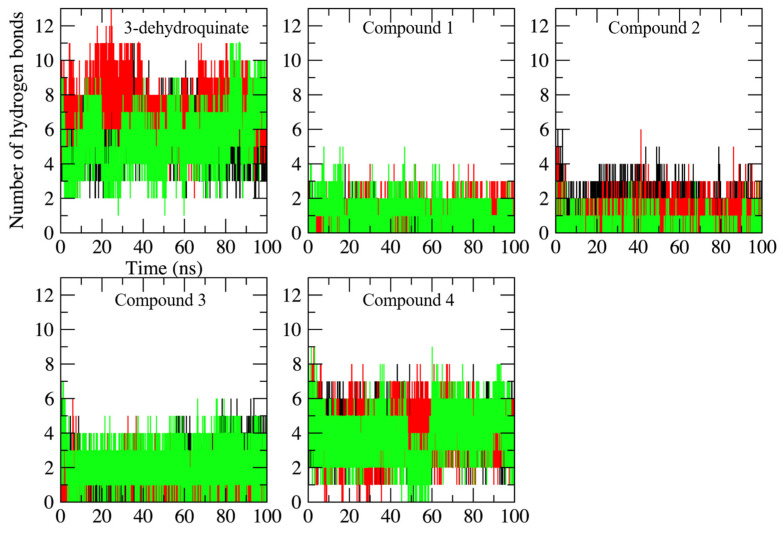
Number of hydrogen bonds formed along the 100 ns of simulated time in each complex. Replicates and axes labels are shown as described in Figure 2.

In respect to hydrogen bond formation, as was expected, none of the compounds made as many hydrogen bonds as the substrate (Figure 6). This is the logic: when the structure of the potential inhibitors is analyzed, the number of hydrogen bond donors or acceptors is limited, contrary to what is observed in the substrate. Therefore, the binding of these compounds was governed (as shown later) by Van der Waals interactions, which are the most common type of interactions and influence the stability of the complex [35].

**Figure 7:**
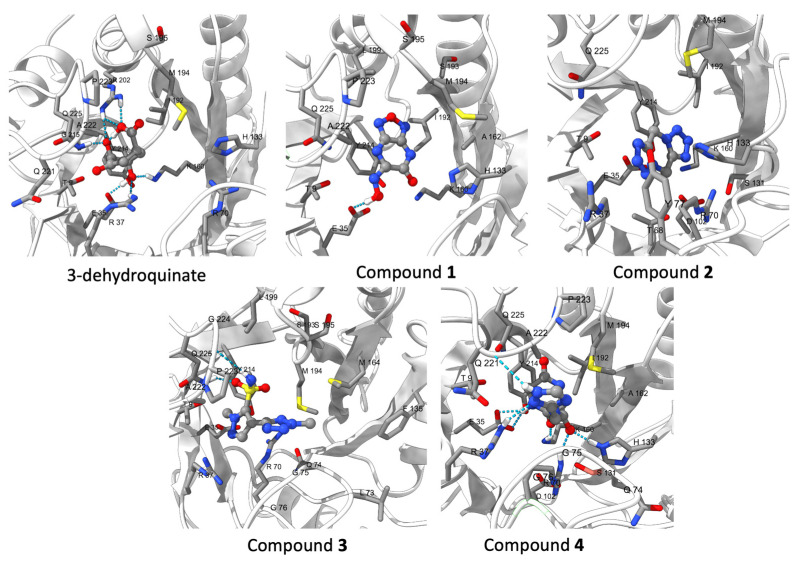
3D interaction map of each complex; the image shows a representative structure. Residues in the binding site were Thr9, Glu35, Arg37, Thr68, Arg70, Asp102, Ser131, His133, Lys160, Ala162, Ile192, Met194, Arg202, Tyr214, Gln221, Ala22, and Gln225 (thin lines). Ligands are on thicker lines at the center of each image. Hydrogen bonds are depicted as blue dashed lines.

According to a representative structure of the most populated cluster obtained from clustering the last 20 ns of each replicate, compound **1** made a hydrogen bond with Glu35, whilst compound **2** did not make any interaction. Compound **3** made hydrogen bonds with Pro223 and Gln225, and compound **4** formed the same type of interaction with Glu35, Arg37, Arg70, His133, Lys160, and Gln225. Some of these interactions were shared by the substrate, such as Glu35, Lys160, and Gln225, additionally, a hydrogen bond with Arg37 and Arg202 was established by the substrate (Figure 7). Therefore, it can be said that compounds **1**, **3**, and **4** were able to make some of the same interactions as the substrate.

### 2.3. Binding Free and Interaction Energies

To study the stability of each complex, binding free energy was calculated using the MM/PBSA approximation. MM/PBSA is a computational way to estimate relative binding affinities at a reduced computational cost that could be used to obtain a qualitative ranking of the compounds tested [36,37,38,39,40]. Even when there are more robust ways to calculate these affinities, such as free energy perturbation [41] or thermodynamic integration [42,43], those methodologies are very time- and resource-consuming. Herein, we used the MM/PBSA method, performing three independent replicas (100 ns) for each complex obtained from the molecular docking screening (also realized in triplicate). Clustering analysis was used to choose 100 random structures from each system, concatenate them, and treat them as a single system (300 structures) using GMXPBSA 2.1 scripts. The energy calculations suggest that the complex with 3-dehydroquinate was stabilized by polar contributions (solvation energy terms), whilst in the case of the four potential inhibitors, their complexes were stabilized through apolar contributions (nonsolvation energy terms) (Table 2). These agree with ligand structure; substrate (3-dehydroquinate) has three alcohols and a carboxyl group that allow it to be an acceptor or donor of hydrogen bonds. On the other hand, compound **1** has only one hydroxyl and one carbonyl group, while compound **2** has no hydrogen donor atoms and, as can be seen in Figure 3, has basically hydrophobic interactions with SaDHQD. Compound **3** possesses a hydrogen bond donor and acceptor atom; however, its binding was dominated by apolar contributions. Finally, compound **4** has three carbonyl groups and a 1, 2, 4-triazole ring that might be acting as hydrogen bond acceptors, but like in the other inhibitors, the hydrophobic interactions were the principal component (Figure 3 and Table 2).

**Figure 8:**
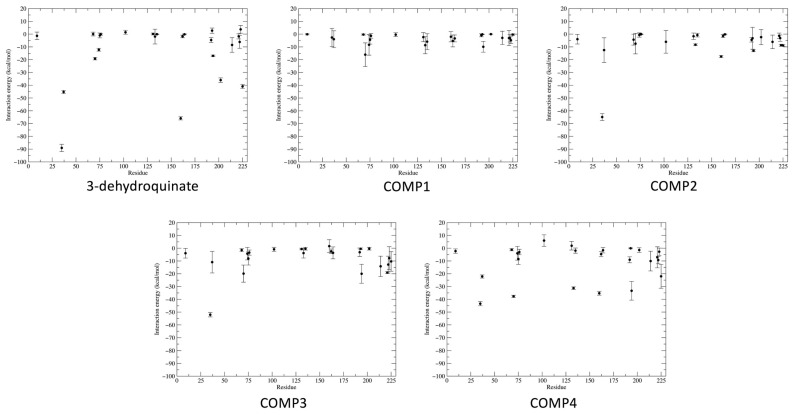
Interaction energy (kcal/mol) between the substrate and each potential inhibitor with residues at 5 Å in the binding site. The residue number and interaction energy values are shown in x and y-axes, respectively. Each point indicates the mean ± SD.

To gain more information about the interactions made between the compounds and the protein through dynamic simulations, interaction energy was estimated for each residue at the binding site. The same 300 frames used for the binding free energy were used to calculate the interaction energy between the corresponding amino acid and compound (Figure 8, Table S2). Additionally, interactions with more than 50% appearance along the simulation time were considered for the analysis (Table 3). As can be observed, the interactions with Arg70, Lys160, Met194, and Ala222 were the only ones shared among potential inhibitors and the substrate crystallographic complex even with the 3-dehydroquinate complex simulated, having additionally high percentages of appearance [19]. The above highlights the importance of these residues as hot spots to interact with the enzyme. Furthermore, from an energetic point of view, compound **4** shows the highest interaction energies at residue level and the highest total interaction energy among the four potential inhibitors. It is important to mention that the total interaction energy includes not only the residues shown in Table 3, but also considers interactions with residues that have less than 50% appearance. 

### 2.4. ADMETox Properties

Finally, an important point in the first steps of the drug design process is to predict the drug-like characteristics of the potential inhibitors. In this context, the ADMETox properties of each compound were analyzed through an in silico approach (Table S3). This analysis was performed considering additional properties to those taken for initial filtering; these included Pharmacokinetics, Druglikeness, and Medicinal Chemistry characteristics. In respect to Pharmacokinetics, compounds **1** and **4** show low GI absorption, whilst in Druglikeness, the best evaluated were compounds **3** and **4** with only one violation of Ghose rules. On the other hand, predictions classified in Medicinal Chemistry are interesting because they involve aspects such as Pan-assay interference compounds (PAINS) and Brenk alerts that analyze the presence of undesirable substructures; synthetic accessibility, a characteristic that becomes important if the molecules reach the lead optimization step; Blood Brain Barrier penetration (BBB); and CYP450 inhibition, among others. From the fifteen characteristics evaluated, none of the compounds received a positive score in all of them; however, the four potential inhibitors approved the majority, highlighting PAINS alerts, synthetic accessibility, BBB, and Pgp inhibition (Table 4). Therefore, considering all the predictions performed, in general, the four compounds meet the commitments to be considered good candidates.

## 3. Materials and Methods

### 3.1. Compound Selection and Filtering

Compound selection was performed from the TRANCHES 3D collection in the ZINC15 database (https://zinc15.docking.org/ accessed on 30 September 2020) [44], which consisted of over 997 million compounds by the time the database was consulted. From this library, compounds with a molecular weight up to 250 Da and a Log *p* value of −1, considering 3-dehydroquinate properties (MW = 198 Da and Log *p* = −1.7), as well as structures without reactive groups (highest reactivity clean), were selected; this process resulted in a database of 123,000 compounds. Thereafter, additional filtering criteria, including physicochemical characteristics in accordance with the Lipinski rule of five, compounds with predicted toxicological parameters (mutagenicity, reproductive, tumorigenic, and irritant effects) were eliminated, structural aspects (such as the number of rotatable bonds <5, number of aromatic rings <4, a polar surface area >75 and <140), and an adequate “druglike” value (−2 to 5) that indicates the potential that a certain compound had in order to become a drug, were applied using Data Warrior V.5.2.1 software (http://www.openmolecules.org/datawarrior/) [45]. Finally, 6700 compounds were selected for the virtual screening process.

### 3.2. Exhaustive Virtual Screening

To perform the virtual screening, the SaDHQD crystal structure (PDBID: 1SFJ, chain A) was used as a receptor [19]. The structure was prepared by removing the crystallographic ligand 3-dehydroquinate, and energy was minimized with 100 steps using the steepest descent method and 10 steps using conjugate gradient as implemented in Chimera UCSF [46]. Compounds were processed with Openbabel 3.1.1 [47] to convert the SDF library to individual pdb files, and then each pdb file was converted to a pdbqt file using prepare_ligand4.py as distributed on MGLTools 1.5.7 [48]. Molecular docking was performed using Autodock Vina [49]. The crystallographic ligand was used as the center of the grid box (104.40, −17.89, −107.70) with a size of 15 × 15 × 15 Å^3^. To validate docking results, a crystallographic ligand was redocked, obtaining an RMSD value of 2.35 ± 0.00 Å. To consider a correct molecular docking study, the RMSD between the crystallographic and the redocked ligand must be lower than 2 Å. Our study had a higher RMSD value, but it is important to point out that we are not doing covalent docking, which is the case for 3-dehydroquinate. Nonetheless, the number and type of interactions found on the crystal and the redocked ligand remain at 82%. Only three interactions were missing on the redocked 3-dehydroquinate (Thr68, Asp102, and Ser131). Three independent docking experiments were made on each ligand (6700 × 3), and all outputs were clustered by binding score. Ligands with a binding score lower than the average minus two standard deviations (4% of the normal distribution) were selected for molecular dynamics simulations (Figure 1).

### 3.3. Exhaustive Molecular Dynamics Simulations

Complexes generated on Autodock Vina for each compound and the crystallographic ligand 3-dehydroquinate were used to perform molecular dynamics simulations using GROMACS 2019 [50,51]. To this end, ligand parameters were obtained from the Automated Topology Builder and Repository (ATB) [52]. Each system was immersed in an orthorhombic cell with SPC water molecules and 0.15 M NaCl to neutralize. Every system was energy minimized with 1000 steepest descent steps, followed by 50,000 steps of molecular dynamics on the NVT and NPT ensembles to equilibrate the system. Finally, 100 ns molecular dynamics simulations were performed using a 2 fs timestep, periodic boundary conditions (PBC), Particle Mesh Ewald (PME) for electrostatics, velocity rescale temperature coupling, and Parrinello–Rahman (NPT ensemble) for pressure coupling. Molecular dynamics simulations were carried out in triplicate for a total of 15 systems (1.5 μs total simulation time). The last 20 ns of every system (2000 frames) were used for clustering structures using α-carbons with the gromacs gmx cluster utility. Clustering was made using a cutoff of 1.25 Å with the GROMOS method [53], which allowed that at least 50% of the structures were clustered on the first cluster.

### 3.4. Binding Free Energy

Additionally, binding free interaction energies of 300 random structures (100 structures of each replica) of the most populated cluster (for every replica) were calculated using the Molecular Mechanics/Poisson-Boltzmann Accessible Area (MM-PBSA) approximation [36,37,54]. MM-PBSA binding-free energies were calculated using GMXPBSA 2.1 scripts [55]. Binding free energy was obtained from the equation:

$$\Delta G_{\mathrm{binding}}=G_{\mathrm{complex}}-\left(G_{\mathrm{protein}\;\mathrm{in}\;\mathrm{complex}}+G_{\mathrm{ligand}\;\mathrm{in}\;\mathrm{complex}}\right)$$

Each free energy value was calculated as follows:

$$<G>=E_{MM}+G_{SOLV}-T<S_{MM}>$$

Entropy was not calculated; therefore, binding free energies reported are the enthalpic contribution to the total binding free energy.

### 3.5. ADMETox Properties

ADMETox properties prediction (Absorption, Distribution, Metabolism, Excretion, and Toxicity) of the selected compounds was carried out using the SwissADME [56] and PREADMET online servers [57] (Bioinformatics and Molecular Design Research Center; 2004). Different parameters, including gastrointestinal absorption, permeability of the blood-brain barrier, potential substrate or inhibitor of G-glycoprotein, inhibitor of the cytochrome family, and artificial synthesis accessibility, were estimated.

## 4. Conclusions

In the present work, a computer-aided drug design strategy was applied to report potential noncovalent SaDHQD inhibitors. After an exhaustive virtual screening protocol, four compounds were selected and characterized (**ZINC000005753647** (**1**), **ZINC000001720488** (**2**), **ZINC000082049768** (**3**), and **ZINC000644149506** (**4**)). Clustering molecular dynamics show that these molecules were able, potentially, to interact with residues important for substrate binding and catalysis when compared to the crystallographic ligand. The interaction energy calculation for each residue in the binding site helped to detect hotspots for potential enzyme inhibition. Finally, the ADMETox properties prediction suggests that these compounds can be considered good candidates. Therefore, the four compounds reported here are excellent options to be considered for future in vitro studies to design new SaDHQD noncovalent inhibitors and contribute to the search for new drugs against MRSA.

## Figures and Tables

**Table 1:** Autodock Vina docking score for the best ligands.

Ligand	Docking Score (kcal/mol)	Structure
3-dehydroquinate	−6.1 ± 0.0	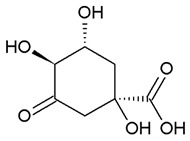
Compound **1**	−7.5 ± 0.0	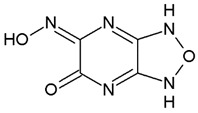
Compound **2**	−7.5 ± 0.0	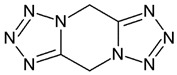
Compound **3**	−7.5 ± 0.0	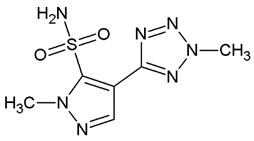
Compound **4**	−7.8 ± 0.0	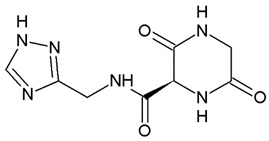

**Table 2:** Binding free energy from potential SaDHQD inhibitors.

Ligand	Total (kcal/mol)	Polar Contribution	Apolar Contribution
3-dehydroquinate	0.00 ± 0.00	0.00 ± 0.00	0.00 ± 0.00
Compound **1**	86.88 ± 8.96	219.17 ± 7.68	−132.29 ± 4.61
Compound **2**	68.67 ± 9.39	218.77 ± 8.51	−150.10 ± 4.04
Compound **3**	29.08 ± 9.33	237.14 ± 8.41	−208.06 ± 4.09
Compound **4**	4.80 ± 8.90	172.73 ± 7.85	−168.03 ± 4.15

Note: Compound binding energies were referenced with respect to substrate binding energies. Positive energy values in compounds mean that the substrate has a higher value (more negative) than the respective compound. On the other hand, a negative energy value means that compounds have a higher value than the substrate.

**Table 3:** Protein/ligand interaction energy over structures used for MM/PBSA analysis.

	3-dehydroquinate	Compound 1	Compound 2	Compound 3	Compound 4
THR9 *	−1.35 ± 2.96 (88.67)		−3.97 ± 3.75 (65.33)	−3.93 ± 3.77 (89.33)	−2.28 ± 1.71 (73.67)
GLU35 *	−89.04 ± 48.34 (88.67)		64.91 ± 47.03 (67.00)	−52.03 ± 29.74 (88.33)	−43.42 ± 27.92 (85.67)
ARG37 *	−45.26 ± 21.24 (88.67)		−12.50 ± 9.63 (67.00)	−10.91 ± 8.45 (89.33)	−22.15 ± 21.41 (72.67)
THR68 *	0.08 ± 1.48 (52.67)		−4.34 ± 4.27 (63.00)		
ARG70 *	−19.21 ± 16.73 (71.67)	−16.05 ± 9.22 (89.33)	−7.47 ± 8.01 (74.33)	−19.87 ± 6.70 (89.33)	−37.71 ± 15.75 (86.00)
**GLN74**		−8.36 ± 8.11 (86.67)		−4.28 ± 4.86 (59.00)	−3.99 ± 5.35 (59.33)
**GLY75**		−4.38 ± 3.40 (83.67)		−8.05 ± 5.10 (89.33)	−8.45 ± 4.22 (85.67)
**GLY76**				−3.42 ± 2.00 (86.33)	−2.98 ± 2.13 (67.33)
ASP102 *			−6.03 ± 8.87 (57.00)		5.97 ± 4.50 (57.67)
SER131 *					1.93 ± 3.36 (78.33)
HIS133 *		−8.65 ± 6.69 (97.33)		−3.83 ± 3.84 (61.67)	−31.24 ± 16.88 (86.00)
**PHE135**		−5.86 ± 6.24 (70.00)			−1.90 ± 2.03 (62.33)
LYS160 *	−65.90 ± 21.63 (88.67)	−2.07 ± 3.98 (58.67)	−17.53 ± 14.40 (68.67)	1.60 ± 5.04 (62.67)	−35.25 ± 25.62 (86.00)
ALA162 *	−1.81 ± 1.02 (67.00)	−5.34 ± 4.63 (80.00)		−2.42 ± 1.81 (58.33)	−4.63 ± 1.99 (86.00)
**MET164**		−3.36 ± 2.73 (63.00)			
ILE192 *	−4.65 ± 1.96 (88.00)		−4.46 ± 1.73 (83.00)		−9.06 ± 2.36 (86.00)
**SER193**	2.73 ± 2.05 (59.33)				
MET194 *	−16.97 ± 10.48 (87.33)	−9.89 ± 4.22 (98.67)	−12.99 ± 13.97 (91.33)	−19.99 ± 7.40 (89.33)	−33.32 ± 7.31 (86.00)
ARG202 *	−35.99 ± 29.86 (76.00)				
TYR214 *	−8.48 ± 5.76 (88.33)		−6.06 ± 5.32 (91.67)	−14.17 ± 7.95 (89.33)	−10.07 ± 7.73 (86.00)
GLN221 *				−19.07 ± 13.41 (73.67)	−7.14 ± 8.21 (55.33)
ALA222 *	−6.12 ± 4.98 (60.67)	−3.28 ± 2.88 (61.33)	−3.12 ± 2.48 (56.67)	−12.81 ± 6.59 (86.67)	−9.28 ± 2.89 (86.00)
**PRO223**	3.71 ± 3.00 (55.67)	−4.76 ± 2.70 (83.33)			−2.74 ± 2.81 (83.33)
GLN225 *	−40.92 ± 27.09 (72.67)		−8.89 ± 11.19 (72.67)	−10.35 ± 7.63 (89.33)	−22.02 ± 9.30 (86.00)
**Total** **interaction energy**	−345.01 ± 12.26	−90.67 ± 22.37	−181.24 ± 21.01	−202.39 ± 23.87	−284.03 ± 19.98

Note: Protein/ligand interaction energy was calculated from any atom of the ligand to any residue of the protein within a 5 Å radius. Only interactions with more than 50% appearance are shown (number in parenthesis). Residues identified in the crystallographic complex are highlighted (*).

**Table 4:** The most relevant ADMETox predicted properties from the SaDHQD potential inhibitors.

Parameter	Compound 1	Compound 2	Compound 3	Compound 4
**Pharmacokinetics**				
GI absortion	Low	High	High	Low
Log K_p_ (Skin permeation)	−7.36 cm/s	−8.33 cm/s	−8.27 cm/s	−8.90 cm/s
**Druglikeness**				
Ghose	No; 2 violations: WLOGP < −0.4, #atoms < 20	No; 3 violations: WLOGP < −0.4, MR < 40, #atoms < 20	No; 1 violation: WLOGP < −0.4	No; 1 violation: WLOGP < −0.4
Veber	Yes	Yes	Yes	Yes
Egan	Yes	Yes	Yes	Yes
Muegge	No; 2 violations: MW < 200, #C < 5	No; 2 violations: MW < 200, #C < 5	Yes	Yes
Bioavailability score	0.55	0.55	0.55	0.55
**Medicinal chemistry**				
PAINS	0 alert	0 alert	0 alert	0 alert
Brenk	3 alerts: imine_1, oxime_1, oxygen-nitrogen_single_bond	0 alert	0 alert	1 alert: beta_keto_anhydride
Leadlikeness	No; 1 violation: MW < 250	No; 1 violation: MW < 250	No; 1 violation: MW < 250	No; 1 violation: MW < 250
Synthetic accesibility	3.60	2.42	2.78	2.61
BBB	0.31	0.08	0.05	0.05
In vitro Caco2 cell permeability	3.94	0.73	1.28	6.54
In vitro CYP 2C19 inhibition	Inhibitor	Inhibitor	Non	Inhibitor
In vitro CYP 2C9 inhibition	Non	Non	Non	Non
In vitro CYP 2D6 inhibition	Non	Non	Non	Non
In vitro CYP 2D6 substrate	Non	Non	Non	Non
In vitro CYP 3A4 inhibition	Non	Non	Non	Inhibitor
In vitro CYP 3A4 substrate	Weakly	Non	Substrate	Non
HIA	40.23	70.25	70.86	27.24
MDCK	1.42	0.72	0.60	0.59
Pgp inhibition	Non	Non	Non	Non
Plasma Protein Binding	2.68	14.37	60.17	5.91

Note: All values were calculated with the SwissADME web tool and PreADMET server. Skin Permeability: in vitro skin permeability-transdermal delivery (logKp, cm/h), Ghose, Veber, Egan, and Muegge (Filters that determine the druglikeness of a compound: no violations are considered ideal). Bioavailability score: it predicts the probability of a compound having at least 10% oral bioavailability in rats, Number of Brenk alerts and PAINS alerts (number of alerts for undesirable substructures/substructures; a result with no alerts is ideal). Leadlikeness: molecules are evaluated according to three parameters: ≤250 MW ≤350, XLOGP ≤3.5, and number of rotatable bonds ≤7; there should be no violations; Synthetic accessibility: refers to the ease of chemical synthesis from 1 (very easy) to 10 (very difficult). BBB: in vivo blood-brain barrier penetration (C.brain/C.blood); Caco2: in vitro Caco−2 cell permeability (nm/s); values > 500 nm s^−1^ indicate a good permeability, and values < 25 nm s^−1^ indicate a low permeability; CYP 2C19 inhibition: in vitro Cytochrome P450 2C19 inhibition; CYP 2C9 inhibition: in vitro Cytochrome P450 2C9 inhibition; CYP 2D6 inhibition: in vitro Cytochrome P450 2D6 inhibition; CYP 2D6 substrate: in vitro Cytochrome P450 2D6 substrate; CYP 3A4 inhibition: in vitro Cytochrome P450 3A4 inhibition; CYP 3A4 substrate: in vitro Cytochrome P450 3A4 substrate; HIA: Human intestinal absorption (HIA, %); a high intestinal abortion percentage is desirable, as indicated by values closest to 100%; MDCK: in vitro MDCK (Mandin Darby Canine Kidney) cell permeability (nm/s), values > 500 nm s^−1^ indicate a good permeability, and values < 25 nm s^−1^ indicate a low permeability; Pgp inhibition: in vitro P-glycoprotein inhibition; Plasma Protein Binding: in vitro plasma protein binding (%); a value of >90% is desirable.

## Data Availability

Not applicable.

## Supplementary Materials

The following supporting information can be downloaded at: https://www.mdpi.com/article/10.3390/ph16081148/s1, Table S1: Molecular docking scores from the three independent runs: Excell_datasheet; Table S2: Interaction energy (kcal/mol) between ligands and those residues at 5 Å. Table S3: ADMETox predicted properties from the SaDHQD potential inhibitors.
