# Supplementary material for: Discovery of Potential Noncovalent Inhibitors of Dehydroquinate Dehydratase from Methicillin-Resistant Staphylococcus aureus through Computational-Driven Drug Design
Source: Pharmaceuticals (Basel). 2023 Aug 12;16(8):1148. doi: 10.3390/ph16081148 (PMC10458038; doi:10.3390/ph16081148)
Supplement: Supplementary file 1 [file pharmaceuticals-16-01148-s001.zip › Table S2.pdf]

Table S2. Interaction energy (kcal/mol) between ligands and those residues at 5 Å

Residue	3-dehydroquinate	Compound 1	Compound 2	Compound 3	Compound 4
9	-1.35+/-2.96	-0.12+/-0.43	-3.97+/-3.75	-3.93+/-3.77	-2.28+/-1.71
35	-89.04+/-2.79	-2.63+/-7.11	-64.91+/-2.72	-52.03+/-1.72	-43.42+/-1.61
37	-45.26+/-1.23	-3.99+/-6.63	-12.50+/-9.63	-10.91+/-8.45	-22.15+/-1.24
68	0.08+/-1.48	-0.32+/-0.44	-4.34+/-4.27	-1.48+/-1.09	-1.21+/-0.86
70	-19.21+/-0.97	-16.05+/-9.22	-7.47+/-8.01	-19.87+/-6.70	-37.71+/-0.91
74	-12.23+/-1.09	-8.36+/-8.11	-0.69+/-1.68	-4.28+/-4.86	-3.99+/-5.35
75	-1.02+/-1.92	-4.38+/-3.40	-0.10+/-0.36	-8.05+/-5.10	-8.45+/-4.22
76	-0.19+/-0.61	-1.34+/-1.87	-0.09+/-0.10	-3.42+/-2.00	-2.98+/-2.13
102	1.42+/-1.55	-0.42+/-1.45	-6.03+/-8.87	-0.86+/-1.63	5.97+/-4.50
131	0.13+/-0.72	-2.37+/-3.53	-1.67+/-2.52	-0.63+/-0.65	1.93+/-3.36
133	-1.92+/-5.79	-8.65+/-6.69	-8.29+/-0.69	-3.83+/-3.84	-31.24+/-0.97
135	-0.08+/-0.16	-5.86+/-6.24	-0.76+/-1.45	-0.44+/-1.03	-1.90+/-2.03
160	-65.90+/-1.25	-2.07+/-3.98	-17.53+/-0.83	1.60+/-5.04	-35.25+/-1.48
162	-1.81+/-1.02	-5.34+/-4.63	-1.51+/-1.12	-2.42+/-1.81	-4.63+/-1.99
164	-0.18+/-0.33	-3.36+/-2.73	-0.20+/-0.30	-3.64+/-4.67	-1.58+/-2.12
192	-4.65+/-1.96	-0.95+/-1.17	-4.46+/-1.73	-3.16+/-3.26	-9.06+/-2.36
193	2.73+/-2.05	-0.16+/-0.14	-3.24+/-8.53	-0.47+/-0.63	-0.08+/-0.34
194	-16.97+/-0.61	-9.89+/-4.22	-12.99+/-0.81	-19.99+/-7.40	-33.32+/-7.31
202	-35.99+/-1.72	-0.02+/-0.06	-2.37+/-5.95	-0.38+/-1.04	-1.43+/-1.80
214	-8.48+/-5.76	-2.93+/-5.17	-6.06+/-5.32	-14.17+/-7.95	-10.07+/-7.73
221	-1.76+/-1.99	-3.07+/-5.89	-1.35+/-2.17	-19.07+/-0.77	-7.14+/-8.21
222	-6.12+/-4.98	-3.28+/-2.88	-3.12+/-2.48	-12.81+/-6.59	-9.28+/-2.89
223	3.71+/-3.00	-4.76+/-2.70	-8.70+/-0.80	-7.80+/-8.94	-2.74+/-2.81
225	-40.92+/-1.56	-0.35+/-0.54	-8.89+/-0.65	-10.35+/-7.63	-22.02+/-9.30
