# Supplementary material for: Discovery of Potential Noncovalent Inhibitors of Dehydroquinate Dehydratase from Methicillin-Resistant Staphylococcus aureus through Computational-Driven Drug Design
Source: Pharmaceuticals (Basel). 2023 Aug 12;16(8):1148. doi: 10.3390/ph16081148 (PMC10458038; doi:10.3390/ph16081148)
Supplement: Supplementary file 1 [file pharmaceuticals-16-01148-s001.zip › Table S3.pdf]

Table S3. ADMETox predicted properties from the SaDHQD potential inhibitors.

Parameter	**Compound 1**	**Compound 2**	**Compound 3**	**Compound 4**
Molecular weight	169.10 g/mol	164.13 g/mol	243.25 g/mol	238.20 g/mol
Fraction Csp3	0.00	0.50	0.33	0.38
Num rotatable bonds	0	0	2	4
Num H-bond acceptors	6	6	7	5
Num. H-bond donors	3	0	1	4
Molar refractivity	50.27	34.25	52.56	60.10
TPSA	107.67 Å^2	87.20 Å^2	129.96 Å^2	128.87 Å^2
		**Lipophilicity**		
Consensus Log $P_{o/w}$	-0.79	-0.84	-1.16	-1.88
		**Water solubility**		
Log S (ESOL)	-0.86	-0.56	-1.24	-0.25
Class	Very soluble	Soluble	Very soluble	Very soluble
		**Pharmacokinetics**		
GI absorption	Low	High	High	Low
Log K_P (Skin permeation)	-7.36 cm/s	-8.33 cm/s	-8.27 cm/s	-8.90 cm/s
		**Druglikeness**		
Lipinski	Yes; 0 violation	Yes; 0 violation	Yes; 0 violation	Yes; 0 violation
Ghose	No; 2 violations: WLOGP<-0.4, #atoms<20	No; 3 violations: WLOGP<-0.4, MR<40, #atoms<20	No; 1 violation: WLOGP<-0.4	No; 1 violation: WLOGP<-0.4
Veber	Yes	Yes	Yes	Yes
Egan	Yes	Yes	Yes	Yes
Muegge	No; 2 violations: MW<200, #C<5	No; 2 violations: MW<200, #C<5	Yes	Yes
Bioavailability score	0.55	0.55	0.55	0.55
		**Medicinal chemistry**		
PAINS	0 alert	0 alert	0 alert	0 alert
Brenk	3 alerts: imine_1, oxime_1, oxygen-nitrogen_single_bond	0 alert	0 alert	1 alert: beta_keto_anhydride
Leadlikeness	No; 1 violation: MW<250	No; 1 violation: MW<250	No; 1 violation: MW<250	No; 1 violation: MW<250

Synthetic accesibility	3.60	2.42	2.78	2.61
BBB	0.31472	0.0823177	0.0587745	0.0524834
in vitro Caco2 cell permeability	3.94346	0.73613	1.28144	6.54818
in vitro CYP 2C19 inhibition	Inhibitor	Inhibitor	Non	Inhibitor
in vitro CYP 2C9 inhibition	Non	Non	Non	Non
in vitro CYP 2D6 inhibition	Non	Non	Non	Non
in vitro CYP 2D6 substrate	Non	Non	Non	Non
in vitro CYP 3A4 inhibition	Non	Non	Non	Inhibitor
in vitro CYP 3A4 substrate	Weakly	Non	Substrate	Non
HIA	40.239613	70.251543	70.866318	27.244666
MDCK	1.42037	0.720713	0.602251	0.597793
Pgp inhibition	Non	Non	Non	Non
Plasma Protein Binding	2.683644	14.374404	60.179920	5.910365

*All values were calculated with SwissADME web tool and PreADMET server. Molecular weight (Default Range 50-500 Da), Fraction Csp3: Ratio of sp^3 hybridized carbons over the total carbon count of the molecule (at least 0.25), number of rotatable bonds (0-5), number of hydrogen acceptors (0-10), number of hydrogen donors (0-5), Molar refractivity (40-130), TPSA: topological polar surface area (20-130), ESOL (calculates Log S property) values should not exceed 6, octanol/water partition coefficient (consensus LOGP: -2 to 10), Lipinski, Ghose, Veber, Egan and Muegge (Filters that determine druglikeness of a compound: no violations are considered ideal), Bioavailability score: it predicts the probability of a compound to have at least 10% oral bioavailability in rat, Number of Brenk alert and PAINS alert (number of alerts for undesirable substructures/substructures, a result with No alerts is ideal), Leadlikeness: Molecules are evaluated according to three parameters: ≤250 MW ≤350, XLOGP ≤3.5 and number of rotatable bonds ≤7, there should be no violations; Synthetic accesibility: refers to easyness of chemical synthesis from 1(very easy) to 10 (very difficult). BBB: *in vivo* blood-brain barrier penetration (C.brain/C.blood), Buffer solubility: Calculated water solubility value in buffer system by SK atomic types (mg/L), Caco2: *in vitro* Caco-2 cell permeability (nm/sec), values > 500 nm sec^{-1} indicate a good permeability and values < 25 nm sec^{-1} indicate a low permeability, CYP 2C19 inhibition: *in vitro* Cytochrome P450 2C19 inhibition, CYP 2C9 inhibition: *in vitro* Cytochrome P450 2C9 inhibition, CYP 2D6 inhibition: *in vitro* Cytochrome P450 2D6 inhibition , CYP 2D6 substrate: *in vitro* Cytochrome P450 2D6 substrate , CYP 3A4 inhibition: *in vitro* Cytochrome P450 3A4 inhibition, CYP 3A4 substrate: *in vitro* Cytochrome P450 3A4 substrate , HIA: Human intestinal absorption (HIA, %), a high intestinal abortion percentage is desirable indicated by values closest to 100%, MDCK: *in vitro* MDCK (Mandin Darby Canine Kidney) cell permeability (nm/sec), values > 500 nm sec^{-1} indicate a good permeability and values < 25 nm sec^{-1} indicate a low permeability, Pgp inhibition: *in vitro* P-glycoprotein inhibition, Plasma Protein Binding: *in vitro* plasma protein binding (%), a value of >90% is desirable, Skin Permeability: *in vitro* skin permeability-transdermal delivery (logKp, cm/hour).
